# Vestibular function assessment in sudden hearing loss^☆^

**DOI:** 10.1016/j.bjorl.2022.04.007

**Published:** 2022-05-20

**Authors:** Nathalia de Paula Doyle Maia, Karen de Carvalho Lopes, Fernando Freitas Ganança

**Affiliations:** Universidade Federal de São Paulo (UNIFESP), Departamento de Otorrinolaringologia e Cirurgia de Cabeça e Pescoço, Ambulatório de Otoneurologia, São Paulo, SP, Brazil

**Keywords:** Vestibular diseases, Sudden hearing loss, Head impulse test, Vestibular evoked myogenic potentials, Caloric testing

## Abstract

**Objectives:**

To perform vestibular assessment using cervical and ocular vestibular evoked myogenic potential, video head impulse test and caloric testing in patients with sudden hearing loss. Moreover, to evaluate the correlation of dizziness with vestibular tests and the correlation of vestibular tests with hearing prognosis.

**Methods:**

This is an observational, longitudinal and prospective study, including participants diagnosed with sudden hearing loss. The participants underwent cervical and ocular vestibular evoked myogenic potential, video head impulse test and caloric testing. The audiometric assessment was performed at the beginning and at the end of the follow-up. A value of *p* ≤ 0.05 was considered statistically significant.

**Results:**

Seventeen patients were included in the study sample, with a mean age of 45.4 ± 11.1 years. Five participants (29.41%) had dizziness and 15 (88.23%) had tinnitus. All participants underwent vestibular evaluation through cervical and ocular vestibular evoked myogenic potential and video head impulse test, and 13 of them were evaluated through caloric testing. The cervical vestibular evoked myogenic potential was considered altered in five (29.41%) participants, while 11 (64.71%) showed alterations at the ocular vestibular evoked myogenic potential. The video head impulse test and the caloric testing were considered altered in seven (41.18%) and five (38.46%) participants, respectively. There was no statistically significant correlation between the clinical data and the results of vestibular tests or hearing recovery, nor between the results of vestibular tests and hearing recovery.

**Conclusion:**

The assessment through vestibular evoked myogenic potential, video head impulse test and caloric testing showed vestibular involvement in some participants. However, it cannot be stated that the results of the vestibular tests are related to the hearing prognosis of sudden hearing loss.

## Highlights

• Vestibular involvement in sudden hearing loss was very frequent (88.23%).
• The ocular vestibular evoked myogenic potential was the most frequently altered vestibular test.
• The vestibular test results were not related to hearing prognosis.

## Introduction

Sudden hearing loss (SHL) is defined as a hearing loss of at least 30 dB, affecting three or more consecutive audiometric frequencies, with sudden onset within 72 h.^1^ Tinnitus is one of the symptoms present in the majority of cases^2^ and vertigo seems to occur in 30%–60% of patients.^2^

In addition to damage to the hearing function, SHL can also compromise vestibular function, which could be explained by the hypothesis of disease extension due to the anatomical proximity of the cochlea, vestibule and semicircular canals.^3^, ^4^ Some complementary tests, such as the cervical vestibular evoked myogenic potential (cVEMP) and the ocular vestibular evoked myogenic potential (oVEMP), video head impulse testing (vHIT) and caloric testing may be useful to identify vestibular system alterations caused by SHL.

It is believed that the prognosis regarding hearing recovery may be related to several factors, such as the patient’s age, presence of vertigo at symptom onset, degree and configuration of the hearing loss observed in the audiometry and the time between hearing loss onset and the treatment.^2^ Studies that performed the vestibular assessment using VEMP,^1^, ^5^, ^6^, ^7^ vHIT^8^ and caloric testing^1^, ^5^, ^9^ in patients with SHL observed that the abnormal results in these tests could also be related to a worse hearing prognosis.

## Methods

This is an observational, longitudinal and prospective study, approved by the Research Ethics Committee of the institution (Opinion n. 2937124/CAAE 97623018.2.0000.5505).

Patients diagnosed with idiopathic unilateral SHL, who met the eligibility criteria, from November 2019 to March 2021 were included in this study. All study participants had undergone oral corticosteroid treatment prior to inclusion in the study (prednisone 60 mg/day or deflazacort 90 mg/day) with maximum dose duration depending on the case evolution, at the discretion of the assistant otorhinolaryngologist. The inclusion criteria were: idiopathic unilateral SHL^2^ and age between 18 and 70 years. The exclusion criteria were: difficulty and/or impossibility of performing cervical rotation; ocular motility reduction/restriction; conductive hearing loss on audiometry or type B and C curves on immitanciometry; other neurotological and neurological diseases concomitant with the diagnosis of SHL. The free and informed consent form was applied and the vestibular tests cVEMP, oVEMP, vHIT and caloric testing were always performed by the same professional, the main researcher of this study.

### Tonal and vocal audiometry

The patients underwent hearing assessment during the follow-up, performed by the same professional and using the AD 28 Interacoustics audiometer and AZ 7 Interacoustics immittanciometer. Pure tone audiometry was performed, evaluating the frequencies of 250, 500, 1000, 2000, 3000, 4000, 6000 and 8000 Hz, and vocal audiometry with assessment of the speech recognition threshold and the speech recognition index (SRI). The degree of the initial hearing loss was classified according to the pure tone average (PTA) at 500 Hz, 1000 Hz, 2000 Hz, and 4000 Hz in: normal hearing (0–25 dB); mild (26 dB–40 dB); moderate (41 dB–60 dB); severe (61 dB–80 dB); and profound (≥81 dB) hearing loss.^10^ Additionally, the audiometric configuration of hearing thresholds was also analyzed and classified as ascending, horizontal, descending light, descending accentuated, descending slope, U-shaped, inverted-U curve, and notch.^11^

### Vestibular evoked myogenic potential

**Figure 1:**
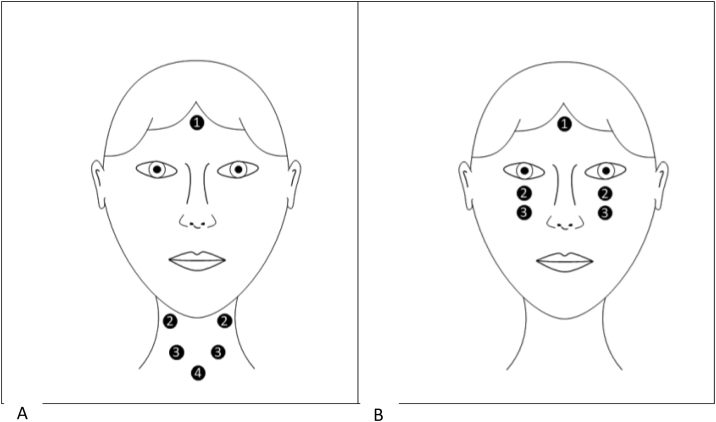
(A) Positioning of electrodes for cVEMP (1- Ground, 2- Active, 3- Electromyography, 4- Reference). (B) Positioning of electrodes for oVEMP (1- Ground, 2- Active, 3- Reference).

The ICS Charter EP 200 equipment (GN Otometrics, Denmark) was used to perform the cVEMP. Adhesive and disposable surface electrodes were used, placed after dermabrasion at the sites as shown in Fig. 1A. The participant remained seated and was instructed to perform a cervical rotation contralateral to the stimulated ear. In oVEMP, the electrodes were positioned as shown in Fig. 1B. The participant remained seated, with the head in the straight position and looking up, reaching a minimum angle of 30°.

Earphones were inserted into the patient’s ear to present the sound stimuli via air conduction. The electrode impedance values ​​remained below 5 kΩ, with a maximum difference of 2 kΩ between them. The potential was assessed with 95 dBHL of intensity. The tests were repeated at least twice to obtain replicable responses. Peaks p13 and n23 were marked for cVEMP and n10 and p15 for oVEMP.^12^, ^13^, ^14^

### Video head impulse test

The ICS impulse equipment (GN Otometrics, Denmark) was used to perform the vHIT. The participant remained seated in a chair, positioned approximately 1.20 m from a fixed target on the wall. After placing the equipment’s glasses on the participant and calibrating them according to the program requirements, the semicircular canals were tested by a head impulse test. During the test, the participants were asked to keep their eyes fixed on the target positioned in front of them. Twenty head impulses were performed for each canal with a velocity of 150–250/s. The vestibulo-ocular reflex (VOR) gain was automatically calculated by the equipment.^15^

### Caloric testing

The ICS Charter EP 200 equipment (GN Otometrics, Denmark) was used to perform the caloric testing. The participant remained seated in a chair positioned at approximately 1.20 m from the light bar. After placing the equipment’s glasses on the participants and calibrating them according to the program requirements, the oculomotricity assessment was performed. Then, the participants lay in the supine position on a stretcher with the head tilted at 30° forward. Eye movements were recorded after stimulation with air at temperatures of 24 °C (cold) and 50 °C (hot), both for 60 s with an air flow rate of 8 L/min. Each ear was evaluated separately, with a 5-min interval between each stimulus. The value of the slow component angular velocity (SCAV) of post-caloric nystagmus was automatically measured by the equipment.^16^

### Outcomes

The cVEMP and oVEMP tests were considered abnormal when the response was absent or asymmetric (AI > 43% in cVEMP and >35.3% in oVEMP).^13^, ^14^ The vHIT was considered altered when the gain in the lateral semicircular and vertical canals was <0.7.^15^ Caloric testing was considered altered when vestibular hypofunction was shown and measured through a labyrinthine predominance (LP) index >19%.^16^

Hearing recovery was assessed based on the hearing gains observed on the PTA and SRI, according to the 2019 SHL Guidelines.^2^ Failure to recover the hearing function was defined by an improvement <10 dB in the PTA. Partial hearing recovery was classified as significant and non-significant recovery, the latter being considered if the degree of initial hearing loss after the SHL rendered the hearing in that ear inoperative (PTA > 50 dB or SRI < 50%). An improvement >10 dB in the PTA or an improvement ≥10% in the SRI in operational ears was considered a significant partial recovery. For ears rendered inoperative by SHL, the return to the useful range of hearing was considered a significant improvement. Complete hearing recovery was defined by a difference <10 dB of the hearing threshold between the affected ear and the unaffected one, associated with a difference of 5%–10% of the SRI between the affected and non-unaffected ears.

### Statistical analysis

The SPSS Statistics software, version 25.0 (IBM Corp., Armonk, NY, USA) was used to perform the statistical analysis. The statistical significance value was set at ≤5% (*p* ≤ 0.05). To analyze the age variable and the degree of hearing loss in relation to the results of vestibular tests, the Student’s *t*-test and Mann–Whitney U-test were used, respectively. The evaluation between these variables and hearing recovery was performed using Spearman’s correlation test.

Fisher’s exact test was used to analyze the variables dizziness, tinnitus and the audiometric configuration of hearing loss in relation to the vestibular test results. To verify the correlation between these variables and hearing recovery, the Kruskal–Wallis and Mann–Whitney U-tests were used.

The Mann–Whitney U-test was used to analyze the association between the vestibular test results and hearing recovery.

## Results

Seventeen participants with idiopathic unilateral SHL were included in the final study sample. The mean age and standard deviation was 45.4 ± 11.1 years, with 9 (52.94%) male and 8 (47.06%) female participants. Hearing loss occurred on the right side in 9 participants (52.94%) and on the left side in 8 (47.06%). Of the total number of assessed participants, 5 (29.41%) had dizziness and 15 (88.23%) had tinnitus ipsilateral to the ear affected by SHL, with all participants having associated dizziness or tinnitus. Four participants had comorbidities, one with systemic arterial hypertension (SAH) and hypothyroidism (participant 12), one with SAH and osteoarthrosis (participant 10), one with SAH (participant 11) and one with osteoarthrosis (participant 16). The demographic and clinical data for each patient are described in Table 1.

**Table 1:** Demographic data, affected ear, presence of dizziness, tinnitus and comorbidities of the participants with sudden idiopathic unilateral hearing loss.

Participant	Gender	Age	Affected ear	Dizziness	Tinnitus	Comorbidities
1	M	32	R	Absent	Present	Absent
2	M	29	L	Absent	Present	Absent
3	F	48	R	Present	Present	Absent
4	M	48	R	Absent	Present	Absent
5	M	24	L	Present	Present	Absent
6	F	43	R	Absent	Present	Absent
7	M	54	L	Absent	Present	Absent
8	F	50	L	Absent	Present	Absent
9	M	38	L	Absent	Present	Absent
10	F	68	R	Absent	Present	Present
11	M	62	R	Absent	Present	Present
12	F	43	L	Present	Absent	Present
13	M	38	L	Absent	Present	Absent
14	F	50	R	Present	Present	Absent
15	F	49	R	Present	Absent	Absent
16	F	51	R	Absent	Present	Present
17	M	45	L	Absent	Present	Absent

M, male; F, female; R, right; L, left.

The initial and final audiometric evaluations were performed, on average, 7 days after the onset of SHL and, on average, 40 days after the initial audiometric evaluation, respectively. The vestibular tests were performed on average 13 days after the onset of SHL (Table 2).

**Table 2:** Period (in days) between the performance of the initial audiometry and vestibular tests in relation to the onset of sudden hearing loss and period (in days) of the performance of the final audiometry in relation to the initial audiometry.

Participant	Performance of the initial audiometry	Performance of the vestibular tests	Performance of the final audiometry
1	2	2	7
2	7	7	55
3	6	7	57
4	3	7	106
5	1	6	28
6	14	21	56
7	4	12	22
8	7	14	105
9	18	24	48
10	9	15	25
11	10	14	18
12	12	25	13
13	16	24	57
14	2	14	28
15	4	9	40
16	2	14	19
17	2	9	7

### Tonal and vocal audiometry

The degree and configuration of the initial and final hearing loss are described in Table 3. Eight participants (47.06%) had hearing loss with PTA > 50 dB and/or SRI < 50% (Participants 3, 4, 5, 8, 10, 14, 15, 17). Two participants had a normal degree according to the PTA; of these, one participant had hearing loss between 2000 Hz and 6000 Hz (participant 2) and another participant had sudden sensorineural hearing loss at the frequencies of 250 Hz, 500 Hz, 6000 Hz and 8000 Hz (participant 16). Although the hearing loss of participant 16 did not meet the SHL criterion initially (only in two consecutive frequencies), approximately two weeks later, this participant showed sudden hearing worsening, being classified in the SHL criterion. Therefore, this participant was included in the study, because inner ear involvement was identified since the initial episode. Hearing recovery was observed in most participants (Table 3): 2 (11.76%) had complete hearing recovery; 11 (64.71%) had partial hearing recovery; and 4 (23.53%) did not have hearing recovery.

**Table 3:** PTA values and initial and final configuration of hearing thresholds and hearing recovery of participants with idiopathic unilateral sudden hearing loss.

Participant	PTA at the initial audiometry	Initial configuration of hearing thresholds	PTA at the final audiometry	Final configuration of hearing thresholds	Hearing recovery
1	Mild	Ascending	Normal	Ascending	Complete
2	Normal	U-shaped	Normal	U-shaped	Did not recover
3	Profound	Horizontal	Severe	Descending accentuated	Partial significant
4	Severe	Ascending	Moderate	U-shaped	Partial significant
5	Severe	Horizontal	Moderate	Horizontal	Partial significant
6	Mild	Slightly descending	Mild	Horizontal	Partial significant
7	Moderate	Inverted U	Mild	Inverted U	Did not recover
8	Severe	Ascending	Moderate	Ascending	Partial significant
9	Moderate	U-shaped	Mild	U-shaped	Partial significant
10	Severe	Horizontal	Normal	Descending light	Partial significant
11	Mild	Horizontal	Normal	Horizontal	Partial significant
12	Moderate	Ascending	Normal	Ascending	Complete
13	Moderate	Ascending	Moderate	Ascending	Did not recover
14	Moderate	Ascending	Mild	Ascending	Partial significant
15	Profound	Horizontal	Normal	Descending-slope	Partial significant
16	Normal	Inverted U	Moderate	Horizontal	Did not recover
17	Severe	Horizontal	Mild	Horizontal	Partial significant

### Vestibular evoked myogenic potential

In the cVEMP, five participants (29.41%) showed no response on the side affected by SHL (participants 7, 11, 14, 16, 17), while oVEMP was altered in 11 (64.71%) participants. Of these, 10 (participants 1, 4, 7, 9, 11, 13, 14, 15, 16, 17) showed no response on the side affected by SHL and one showed asymmetric responses (participant 2), with the response amplitude being lower in the ear affected by SHL (Table 4).

**Table 4:** Results of the video head impulse test, caloric testing, cervical vestibular evoked myogenic potential and ocular vestibular evoked myogenic potential of the participants with idiopathic unilateral sudden hearing loss.

Participant	vHIT	CT	cVEMP	oVEMP
1	Normal	Normal	Normal	Altered
2	Normal	NP	Normal	Altered
3	Normal	Altered	Normal	Normal
4	Normal	Normal	Normal	Altered
5	Normal	Normal	Normal	Normal
6	Altered	Altered	Normal	Normal
7	Normal	Altered	Altered	Altered
8	Normal	Normal	Normal	Normal
9	Altered	Normal	Normal	Altered
10	Altered	Normal	Normal	Normal
11	Normal	Normal	Altered	Altered
12	Altered	Altered	Normal	Normal
13	Altered	Normal	Normal	Altered
14	Altered	Altered	Altered	Altered
15	Normal	NP	Normal	Altered
16	Altered	NP	Altered	Altered
17	Normal	NP	Altered	Altered

vHIT, video head impulse test; CT, caloric testing; cVEMP, cervical vestibular evoked myogenic potential; oVEMP, ocular vestibular evoked myogenic potential; NP, not performed.

### Video head impulse test

All seventeen participants underwent the vHIT. Seven (41.18%) had a decrease in the VOR gain on the side affected by SHL, as described in Table 4. Six participants had a decrease in the VOR gain in the vertical semicircular canals, three of them had reduced gain in the posterior canal (participants 6, 10, 14) and three in the anterior semicircular canal (participants 9, 12, 13). One participant had reduced VOR gain in the lateral and posterior semicircular canals, followed by overt saccades (participant 16).

### Caloric testing

Thirteen participants underwent caloric testing (76.47%). Of these, 5 (38.46%) had altered results due to unilateral hypofunction on the side affected by SHL (participants 3, 6, 7, 12, 14), as described in Table 4.

### Analysis of the association between age, tinnitus, degree and configuration of hearing loss, results of vestibular tests and hearing recovery

There was no statistically significant difference between age, tinnitus, degree and configuration of hearing loss and the vestibular test results. Similarly, no statistically significant correlation was observed between these variables and hearing recovery.

### Analysis of the association between dizziness and the vestibular test results and between dizziness and hearing recovery

No statistically significant association was observed between the presence of dizziness and the vestibular test results (Table 5). Similarly, there was no statistically significant correlation between the presence of dizziness and hearing recovery (*p* = 0.171).

**Table 5:** Analysis of the association between the presence of dizziness and vestibular test results in the participants with idiopathic unilateral sudden hearing loss.

Exam	Conclusion	Dizziness						*p*
		Absent		Present		Total		
		n	%	n	%	n	%	
vHIT	Altered	5	41.67	2	40.00	7	41.18	>0.999
	Normal	7	58.33	3	60.00	10	58.82	
CT	Altered	2	22.22	3	75.00	5	38.46	0.217
	Normal	7	77.78	1	25.00	8	61.54	
cVEMP	Altered	4	33.33	1	20.00	5	29.41	>0.999
	Normal	8	66.67	4	80.00	12	70.59	
oVEMP	Altered	9	75.00	2	40.00	11	64.71	0.280
	Normal	3	25.00	3	60.00	6	35.29	
	Total	12	100	5	100	17	100	

vHIT, video head impulse test; CT, caloric testing; cVEMP, cervical vestibular evoked myogenic potential; oVEMP, ocular vestibular evoked myogenic potential.

### Analysis of the association between the vestibular test results and hearing recovery

There was no statistically significant difference between the vestibular test results and hearing recovery (Table 6).

**Table 6:** Analysis of the association between hearing recovery and vestibular test results in participants with idiopathic unilateral sudden hearing loss.

Test	Conclusion	Hearing recovery								*p*
		Did not recover		Partial		Complete		Total		
		n	%^a^	n	%^a^	n	%a	n	%^b^	
vHIT	Altered	2	28.57	4	57.14	1	14.29	7	41.18	0.905
	Normal	2	20.00	7	70.00	1	10.00	10	58.82	
CT	Altered	1	20.00	3	60.00	1	20.00	5	38.46	>0.999
	Normal	1	12.50	6	75.00	1	12.50	8	61.54	
cVEMP	Altered	2	40.00	3	60.00	0	0.00	5	29.41	0.381
	Normal	2	16.67	8	66.67	2	16.67	12	70.59	
oVEMP	Altered	4	36.36	6	54.55	1	9.09	11	64.71	0.161
	Normal	0	0.00	5	83.33	1	16.67	6	35.29	
	Total	4	23.53	11	64.71	2	11.76	17	100	

NC, not calculated.

a Calculated from the line total.

b Calculated from the column total.

## Discussion

The presence of dizziness and alterations in the vestibular tests, such as caloric testing, vHIT, and VEMP, have been associated with a worse prognosis in SHL.^2^, ^8^, ^17^ However, there is no consensus yet in the literature on the prognostic factors for SHL.^2^, ^18^ In addition to cochlear symptoms, vestibular system impairment may occur in SHL,^19^ as was observed in the participants of the present study due to the presence of dizziness and vestibular test alterations. However, it is important to emphasize that vestibular involvement may be present in patients with SHL even in the absence of dizziness.^19^

Regarding the possible prognostic factors related to SHL, the literature data are conflicting. Although some retrospective studies have suggested that older age, presence of tinnitus and more pronounced hearing loss could be factors of worse prognosis for hearing recovery,^18^, ^20^, ^21^ none of these clinical data evaluated in the present study showed a correlation with hearing recovery. The findings of the current study are supported by other investigations in the literature that have also evaluated hearing recovery in SHL.^22^, ^23^, ^24^, ^25^

In the present study, most of the participants (76.47%) were aged between 30 and 60 years, which is, therefore, in agreement with the literature regarding the age of highest incidence of SHL.^24^ Studies have suggested that aging may be related to the functional impairment of vestibular receptor cells, compromising vestibular tests.^26^, ^27^ Although this fact was taken into account in the present study, it is important to highlight that alterations were found in the vestibular tests of young adults, while normal test results were observed in the elderly. Therefore, the findings of the vestibular tests in this study seem to be associated with SHL. The presence of dizziness was more frequent in ears with altered caloric testing and with normal cVEMP, oVEMP and vHIT results, although these findings were not statistically significant. Moreover, even in the absence of dizziness, 83.33% of the participants showed alterations in at least one of the vestibular tests. Thus, the absence of dizziness in participants with SHL does not seem to be a clinical information that allows inferring the absence of vestibular system impairment and vestibular test alterations. Consistent with the findings of the present study, Hong et al. observed cVEMP alterations in patients with SHL without associated dizziness, hence suggesting that the vestibular system, particularly the saccule, would have a subclinical involvement in patients with SHL.^28^ It was also observed that an altered cVEMP was not related to the hearing prognosis.^28^ The authors hypothesized that the saccule involvement would occur due to the anatomical proximity to the cochlea, similarity between the structures of the cochlear and vestibular hair cells, and due to the common vascularization between the cochlea and the posterior labyrinth.^28^ Khetarpal investigated the histopathological characteristics of the temporal bone (semicircular canals, saccule, utricle, and vestibular ganglion) in SHL patients with and without dizziness and found no correlation between the presence of this symptom and damage to the assessed structures.^29^ Therefore, it has been suggested that vestibular symptoms concomitant with SHL could be caused by ultrastructural alterations in the vestibular nerves and sensory cells or by alterations in their biochemical environment.^29^

In the current study, vestibular involvement in SHL was very frequent, approximately 88.23%, as demonstrated by the altered vestibular tests in most participants. The oVEMP was the most frequently altered vestibular test when compared to vHIT, caloric testing and cVEMP. This finding is in agreement with some studies,^3^, ^30^, ^31^ and the more frequent utricular involvement could be explained by the fact that the bone canal of the superior vestibular nerve is longer and narrower and, thus, may be more susceptible to ischemic alterations.^30^

Regarding the final hearing evaluation, it was observed that all participants who showed complete hearing recovery had their hearing improvement observed within the first two weeks of the onset of SHL. According to these findings, even though the follow-up should be prolonged for a better assessment of patients with SHL, it is suggested that complete hearing recovery may preferably occur within the first weeks after SHL.

The present study observed that all participants who did not show hearing recovery had alterations in the oVEMP test. As for the cVEMP, vHIT and caloric testing, it was observed that most participants with altered results also did not show complete hearing recovery. However, no statistically significant association was observed between the presence of alterations in the cVEMP, oVEMP, vHIT or caloric testing and hearing recovery. This fact suggests that the recovery of cochlear function in SHL could, to some extent, be independent of the extent of damage to the vestibular system.

Considering the studies published in the literature, only one, a retrospective study, evaluated patients with SHL associated with dizziness, through the analysis of all the tests discussed in the present study (cVEMP, oVEMP, caloric testing and vHIT) and correlated them with the presence of a vestibular clinical manifestation, spontaneous nystagmus.^32^ The vast majority of patients in the group that did not have associated spontaneous nystagmus did not exhibit any changes in the vestibular function assessment.^32^ However, a predilection for the posterior canal involvement, as demonstrated by the vHIT, was found in patients who had spontaneous nystagmus. However, it is important to emphasize that, unlike the present study, all the participants of the abovementioned study had dizziness (inclusion criterion) and the study did not evaluate the patients’ hearing recovery.

The main limitation of our study is the small number of participants allocated to the subgroups of variables, which could have reduced the power of the statistical analysis. However, based on the performed literature review, this is the first prospective study that evaluated hearing recovery in patients with SHL using a comprehensive vestibular assessment through cVEMP, oVEMP, caloric testing and vHIT. Unlike most studies published in the literature, the criteria defined by the latest clinical practice guideline on SHL^2^ and the updated reference criteria of vestibular tests were used,^13^, ^14^, ^15^, ^16^ which could justify, at least in part, the differences from the findings of other studies. Therefore, it is necessary to carry out multicenter, randomized, controlled trials, with a larger sample size and using updated and consistent criteria from the scientific literature, so that the correlation between dizziness, vestibular test results and hearing recovery can be better evaluated in SHL.

## Conclusion

The vestibular tests cVEMP, oVEMP, vHIT and caloric testing may be altered in patients with idiopathic unilateral SHL, but their results were not related to the hearing prognosis in SHL in the assessed sample.

## Conflicts of interest

The authors declare no conflicts of interest.
